# An Investigation into the Effects of Frailty and Sarcopenia on Postoperative Anesthesia Recovery and Complications Among Geriatric Patients Undergoing Colorectal Malignancy Surgery

**DOI:** 10.3390/medicina61060969

**Published:** 2025-05-23

**Authors:** Rüştü Özdemir, Ferda Yaman

**Affiliations:** Department of Anesthesiology and Reanimation, Faculty of Medicine, Eskişehir Osmangazi University, Eskişehir 26040, Turkey; rustu.ozdemir@ogu.edu.tr

**Keywords:** frailty, sarcopenia, geriatrics, colorectal cancer surgery, ultrasonography, rectus femoris, vastus intermedius, thigh length, anesthesia recovery, postoperative complications

## Abstract

*Backgrounds and Objectives:* In this study, we aimed to assess preoperative frailty among hospitalized patients over 60 undergoing colorectal cancer surgery. We investigated the impacts of frailty and sarcopenia on postoperative recovery, complications, and discharge time, while also identifying a cost-effective, bedside-accessible USG parameter for diagnosing sarcopenia among patients assessed using the “Sonographic Thigh Adjustment Ratio” method. *Materials and Methods:* In this prospective study, we investigated the impacts of frailty and sarcopenia on the postoperative outcomes of 42 geriatric patients (with American Society of Anesthesiologists (ASA) scores of I–III) undergoing colorectal cancer surgery under general anesthesia. Frailty was assessed using the FRAIL scale, and sarcopenia was evaluated using the STAR (sonographic thigh adjustment ratio). Ultrasonographic measurements of rectus femoris and vastus intermedius muscle thicknesses were taken, and thigh lengths (TLs) were recorded. Ratios, including rectus femoris thickness/TL (RFT/TL), vastus intermedius thickness/TL (VIT/TL), and total muscle thickness/TL (TMT/TL), were calculated. Postoperative anesthesia recovery was monitored using the Modified Aldrete Score, indicating the time until discharge from the recovery unit. Complications were classified using the Clavien–Dindo system, and hospital discharge times were noted. *Results:* We observed significant differences between frailty status and ASA scores, as well as between age and frailty status. Muscle thickness significantly differed between the frail and pre-frail patients. Among the sarcopenic patients, age differences were significant. In men, VIT/TL was significantly correlated with sarcopenia diagnosis, whereas, in women, RFT/TL, VIT/TL, and TMT/TL were all correlated with sarcopenia. *Conclusions:* Based on our results, we conclude that VIT/TL measurement can serve as a predictive marker for preoperative sarcopenia, optimizing patient health before surgery.

## 1. Introduction

The increasing elderly population, both in Turkey and worldwide, has led to a rise in the number of surgical procedures performed on the geriatric population. Despite advancements in surgical procedures and anesthetic techniques, elderly patients experience longer hospital stays and have an increased risk of experiencing perioperative complications [1,2]. This phenomenon is particularly prevalent among sarcopenic elderly patients, who have experienced a loss of muscle mass [3]. Colorectal cancer is the most commonly diagnosed malignancy in the gastrointestinal system and represents one of the most frequently curable cancer types following treatment [4]. According to the 2022 Global Cancer Statistics Report, colorectal cancer ranks third in terms of incidence, at 9.6%, and second in terms of cancer-related mortality, at 9.3% [5].

The global prevalence of sarcopenia among individuals over 60 years of age is estimated to be approximately 10%. Sarcopenia predominantly affects the elderly population, with an equal incidence for both males and females [6]. Studies have demonstrated that ultrasonographic measurements of the quadriceps femoris and vastus intermedius muscles in the leg are associated with patients’ nutritional statuses and sarcopenia [7].

Magnetic resonance imaging (MRI) and computed tomography (CT) are considered the gold standards for sarcopenia diagnosis. MRI provides precise measurements of total-body muscle mass [8]. However, the use of MRI is limited by the requirement for expert interpretations and patient transport, a lack of bedside availability, and cost-effectiveness concerns [8,9]. Similarly, CT is limited because of radiation exposure and similar logistical constraints. In contrast, ultrasonography (USG) is portable, cost-effective, and can be performed at the bedside without exposing the patient to radiation. In this method, high-frequency ultrasound probes allow peripheral muscle tissues and their dimensions to be rapidly assessed [10,11]. Recent studies have integrated USG into diagnostic protocols for sarcopenia [12]. While muscle USG has been proposed as an alternative diagnostic method with respect to MRI and CT [13], the International Society of Physical and Rehabilitation Medicine has introduced a new diagnostic algorithm recommending the use of USG over traditional methods for muscle mass assessment [14]. This algorithm records upper-thigh length (TL) by measuring the distance between the greater trochanter of the femur and the knee, as well as anterior thigh muscle thickness via USG. The sonographic thigh adjustment ratio (STAR) is then calculated by dividing the measured muscle thickness (in millimeters) by the body mass index (BMI). A result falling two standard deviations below the reference value is considered indicative of low muscle mass, with threshold values set at 1.0 for women and 1.4 for men [14,15].

Frailty is a clinically identifiable state of heightened vulnerability resulting from age-related declines in physiological systems and functions, leading to impaired resilience to stressors [16,17,18]. Frailty is highly prevalent among the elderly and associated with an increased risk of falls, disability, hospitalization, and mortality [18]. However, there is no universally accepted definition of frailty or method of measuring it in the literature [18,19]. Identifying frailty may allow for more accurate prognostic and surgical risk assessments than conventional methods [20]. Guidelines from the American College of Surgeons, the Association of Anaesthetists of Great Britain and Ireland, and other professional bodies recommend routine preoperative frailty screening [21].

The primary objective of this study was to assess preoperative frailty among hospitalized patients over 60 undergoing colorectal cancer surgery, as well as to investigate the impacts of frailty and sarcopenia on postoperative recovery, complications, and discharge time. We sought to identify a cost-effective, bedside-accessible USG parameter for diagnosing sarcopenia among patients assessed using the STAR method. We aimed to investigate the effects of frailty and sarcopenia on postoperative recovery time, postoperative complications, and the time until hospital discharge. The secondary objective was to find a USG parameter that is cost-effective, easy to apply, rapidly determinable, and feasible for use at the bedside for diagnosing sarcopenia using the STAR method.

## 2. Materials and Methods

After receiving approval from the Non-Interventional Clinical Research Ethics Committee of Eskişehir Osmangazi University (date: 20 June 2023, decision number: 42) (retrospectively registered, clinical Trial registration number: NCT06889714, www.clinicaltrials.gov, Protocol registration and Result System, 17 March 2025), this study was initiated prospectively. All of the patients were informed about the present study before participating and provided verbal and written informed consent.

**Figure 1:**
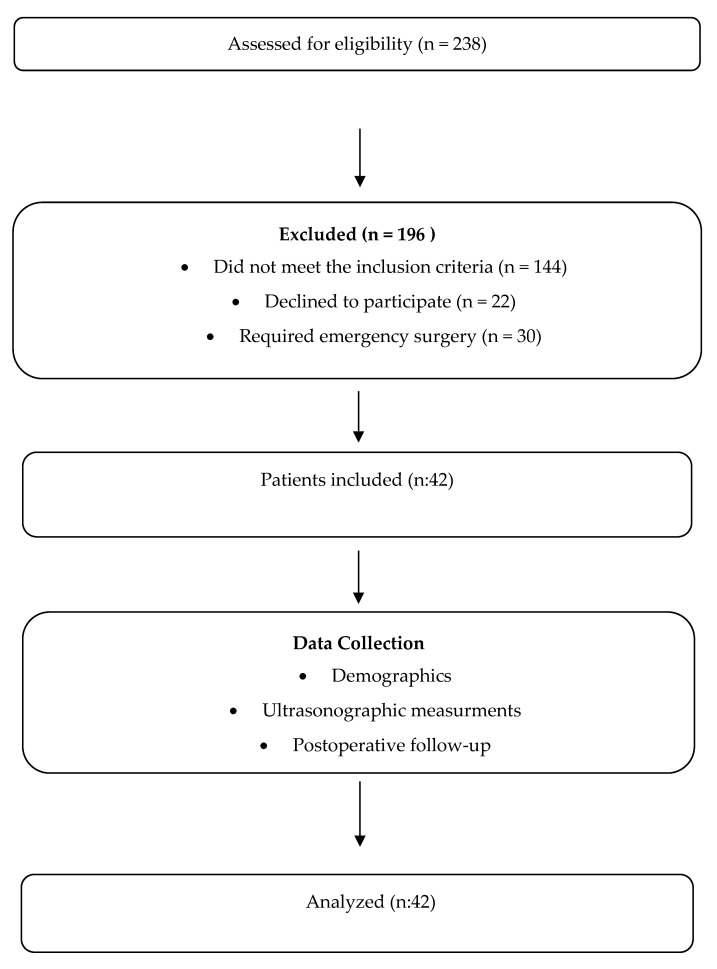
A flowchart depicting the planning phase of this study.

This study included hospitalized patients over 60 years old undergoing colorectal cancer surgery. Preoperative frailty status was assessed using the Fatigue, Resistance, Ambulation, Illness, and Loss of weight (FRAIL) scale [22] (Table 1), while sarcopenia was evaluated by measuring the thickness of the rectus femoris and vastus intermedius muscles via USG [15]. A new measurement method was developed, and the effects of sarcopenia and frailty on anesthesia recovery were analyzed using the Modified Aldrete Score (Table 2), while postoperative complications were classified according to the Clavien–Dindo Classification (CDC) (Table 3). This observational and prospective study included 42 patients, aged 60 years and older, with American Society of Anesthesiologists (ASA) scores of I–III, who were scheduled to undergo colorectal malignancy surgery under general anesthesia. Patients who were classified as having scores of ASA IV–V; those with Parkinson’s disease, Alzheimer’s disease, cerebrovascular disease, neuromuscular disorders, cognitive dysfunction, severe liver disease, heart disease, or kidney disease causing cooperation difficulties; emergency or trauma cases; and those with bilateral lower-extremity amputations were excluded from the present study. Based on the study by Salim et al. [3], a power analysis using G*Power v3.1.9.7 revealed that our study required 42 patients to achieve 95% power. A flowchart outlining our study design, patient selection criteria, and exclusion process is included in Figure 1.

**Figure 2:**
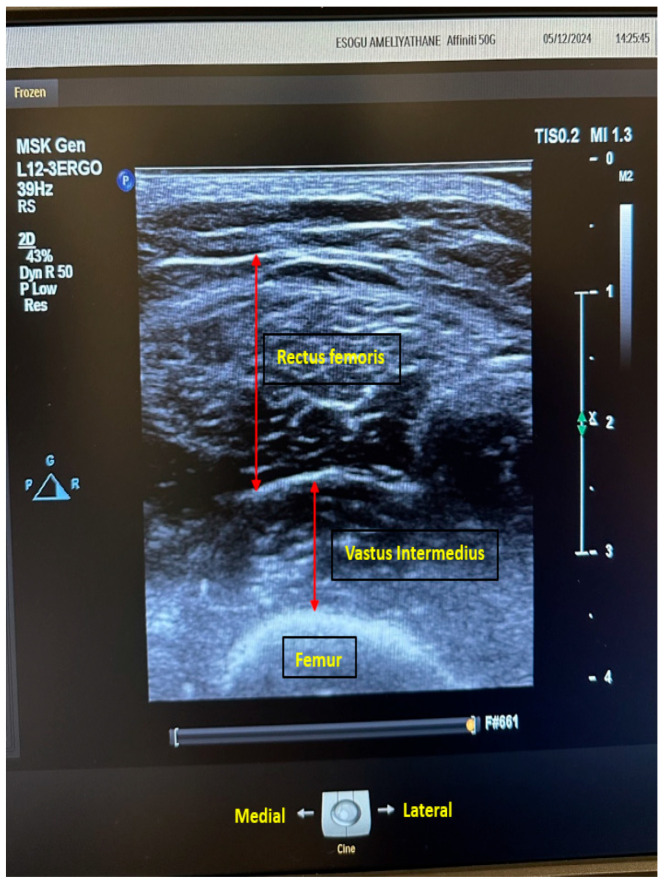
Ultrasonographic image of the thigh: measurement of rectus femoris and vastus intermedius muscle thicknesses (red arrows).

The patients were evaluated preoperatively in the anesthesia clinic. Routine blood tests (accounting for biochemical parameters, coagulation parameters, and complete blood counts) were performed, and physical examinations were conducted. The patients were then transferred to the preoperative waiting room, where demographic data (age, gender, height, body weight, educational status, marital status, and BMI) and ASA scores were recorded. While the patients were in the supine position, we measured and recorded right-thigh length (the distance between the greater trochanter of the femur and the patella). For USG measurements, a linear transducer (Philips Affiniti 50, Philips Medical Systems, Seattle, WA, USA) was used. With an ample amount of ultrasound gel applied, the rectus femoris and vastus intermedius muscles were visualized using the feather-touch technique (Figure 2). Muscle thicknesses were measured and recorded using USG software (9.0.3 Philips Healthcare). Subsequently, the rectus femoris thickness/TL ratio (RFT/TL), vastus intermedius/TL ratio (VIT/TL), and total muscle thickness/TL ratio (TMT/TL) were calculated and recorded. Sarcopenia was assessed using the STAR method. Frailty status was determined using the FRAIL scale, and scores were documented.

For data analysis, The statistical analyses wee performed using MedCalc Statistical Software version 20.009 (MedCalc Software LTD, Ostend, Belgium, https://www.medcalc.org, accessed on 20 November 2024) [23]. Continuous variables are presented as means ± standard deviations, while categorical variables are expressed as percentages. The Shapiro–Wilk test was used to assess the normality of the data distribution. The independent samples *t*-test was used for comparisons between two normally distributed groups, whereas one-way analysis of variance was employed for comparisons between three or more groups. For non-normally distributed data, the Mann–Whitney U test was used for two-group comparisons, and the Kruskal–Wallis H test was applied for comparisons involving three or more groups. Correlation coefficients were determined using Pearson’s correlation for normally distributed variables, and Spearman’s correlation was used for non-normally distributed variables. Pearson’s chi-square and Pearson’s exact chi-square tests were used for the analysis of contingency tables. Receiver operating characteristics analysis was conducted to determine optimal cut-off points and calculate sensitivity and specificity values for independent markers.

## 3. Results

The present study was conducted between 20 July 2023 and 20 July 2024, and included 42 patients who underwent colorectal malignancy surgery under general anesthesia and met the inclusion criteria. The data on these patients were examined prospectively. This study included 29 male and 13 female patients. The youngest patient was 60 years old, while the oldest was 85, with a mean age of 69.8 ± 7.26 years. Table 4 presents the demographic data pertaining to the patients. Table 5 shows a comparison of the patients’ ASA scores with age, height, body weight, BMI, educational status, anesthesia recovery times, CDC grades for postoperative complications, discharge times, sarcopenia status, and frailty status. A statistically significant difference was found between ASA scores and frailty statuses (*p* < 0.05). No statistically significant differences were found for the other parameters (*p* > 0.05).

Table 6 presents the relationships between frailty status and age, gender, BMI, educational status, marital status, postoperative recovery time, discharge time, CDC grade, sarcopenia status, and TMT with respect to the patients studied. A statistically significant difference was found between age and frailty status (*p* < 0.05). Additionally, a statistically significant difference in muscle thickness was found between pre-frailty and frailty statuses (*p* < 0.05). No statistically significant differences were observed for the other parameters (*p* > 0.05).

Table 7 shows the relationships between the patients’ sarcopenia statuses and age, BMI, educational status, marital status, postoperative recovery time, discharge time, and CDC grade. A statistically significant relationship was found between the ages of the male and female sarcopenic patients (*p* < 0.05). No statistically significant differences were observed for the other parameters (*p* > 0.05).

**Figure 3:**
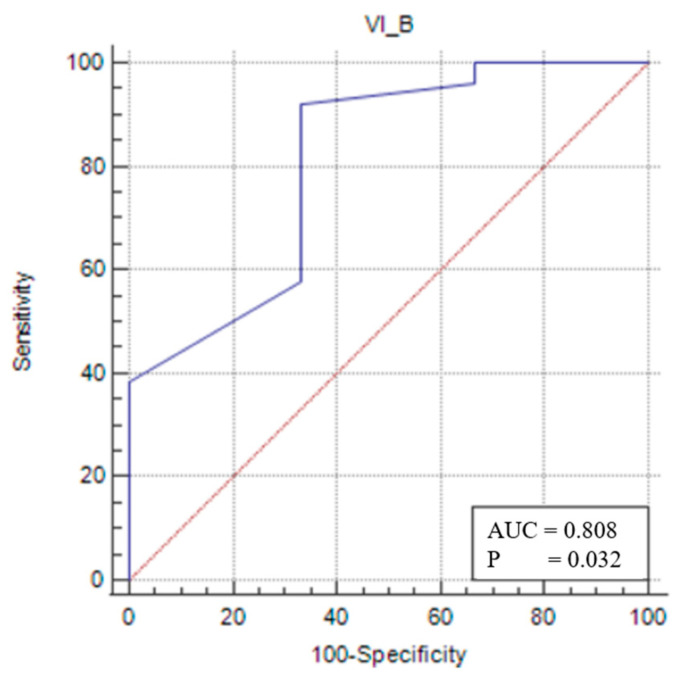
Sensitivity–specificity graph for the vastus intermedius thickness cut-off for male sarcopenic patients. The blue line represents the ROC curve for the variable TOTAL_B in identifying sarcopenia. The diagonal red line indicates the line of no discrimination (AUC = 0.5), serving as a reference.

In the male sarcopenic patients, VIT/TL was found to be statistically significant, and a cut-off value was determined (*p* < 0.05). A VIT/TL value of ≤0.041 (*p* = 0.0319) had 92.31% sensitivity and 66.67% specificity in demonstrating that a patient was sarcopenic (Figure 3).

**Figure 4:**
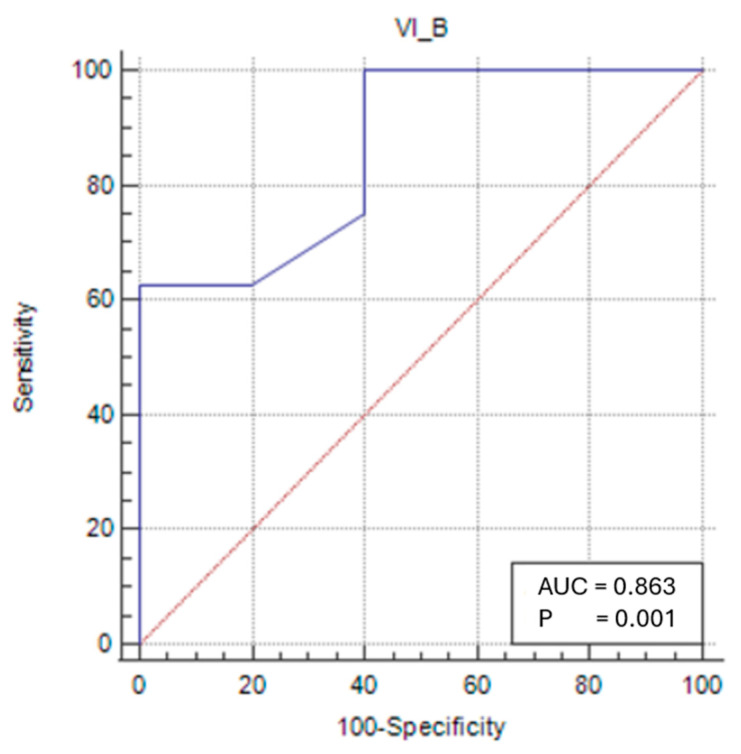
Sensitivity–specificity graph for the vastus intermedius thickness cut-off for female sarcopenic patients. The blue line represents the ROC curve for the variable TOTAL_B in identifying sarcopenia. The diagonal red line indicates the line of no discrimination (AUC = 0.5), serving as a reference.

In the female sarcopenic patients, VIT/TL was found to be statistically significant, and a cut-off value was determined (*p* < 0.05). At a cut-off value of ≤0.028 (*p* = 0.0009), VIT/TL had a sensitivity of 62.5% and a specificity of 100% in predicting sarcopenia status (Figure 4).

**Figure 5:**
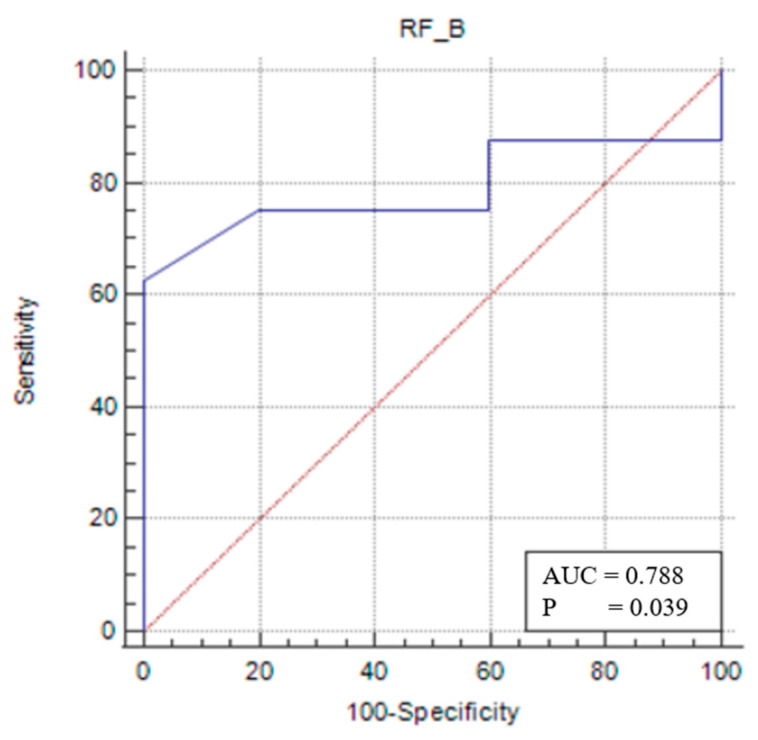
Sensitivity–specificity graph for the rectus femoris thickness cut-off for the female sarcopenic female patients. The blue line represents the ROC curve for the variable TOTAL_B in identifying sarcopenia. The diagonal red line indicates the line of no discrimination (AUC = 0.5), serving as a reference.

For the female sarcopenic patients, an RFT/TL of ≤0.025 (*p* = 0.0391) predicted sarcopenia with 62.5% sensitivity and 100% specificity (Figure 5).

**Figure 6:**
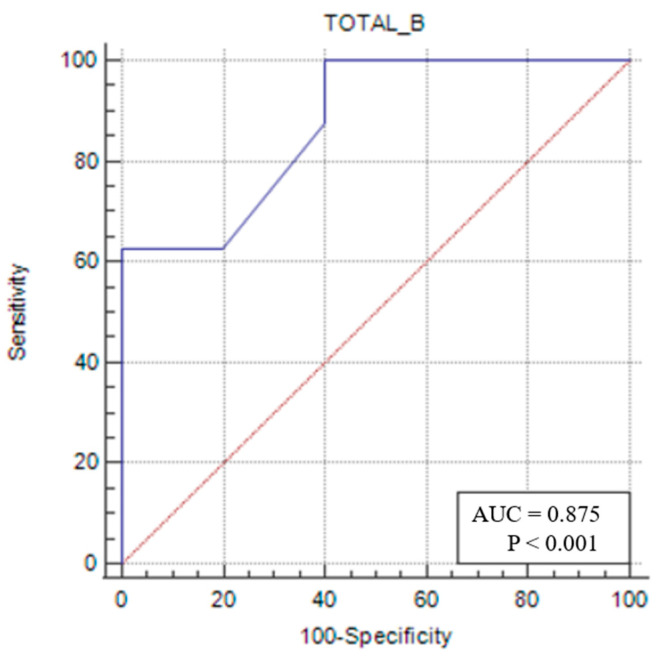
Sensitivity–specificity graph for total muscle thickness cut-off for female sarcopenic patients. The blue line represents the ROC curve for the variable TOTAL_B in identifying sarcopenia. The diagonal red line indicates the line of no discrimination (AUC = 0.5), serving as a reference.

In the same patient group, a TMT/TL of ≤0.053 (*p* = 0.0002) had 62.5% sensitivity and 100% specificity in identifying sarcopenia (Figure 6).

## 4. Discussion

Although a reduction in physiological reserves associated with aging is considered a normal occurrence, frailty represents an extreme manifestation of this condition. All elderly patients are at risk of developing frailty [24,25]. In a meta-analysis conducted by Ofori-Asenso et al. [26], the prevalences of pre-frailty and frailty among adults over 60 years of age were reported to be 15.6% and 4.34%, respectively. Similarly, in a meta-analysis conducted by He et al. [27], 47.4% of hospitalized geriatric patients were identified as frail, while 25.8% were in a pre-frail state. In our study, using the FRAIL scale, we obtained comparable results, identifying 61.9% of the patients as pre-frail and 23.8% as frail. We also observed a statistically significant difference in age between the pre-frail (67.0 (62.3–72.8)) and frail (76.0 (72.8–81.3)) patients.

Studies examining the predictive value of ASA scores and the FRAIL index for postoperative outcomes have demonstrated a correlation between the two [28]. Consistent with the literature, our study revealed a statistically significant association between higher ASA scores and both pre-frailty and frailty.

Various studies have also demonstrated a correlation between frailty and TMT [29,30,31]. Similarly, in our study, we identified a statistically significant difference in total TMT between the pre-frail and frail patients.

A review of the literature reveals that frailty is a key determinant of postoperative surgical outcomes for elderly patients [32,33]. One of the primary objectives of our study was to investigate the impacts of frailty on postoperative anesthesia recovery, complications, and discharge time. However, no statistically significant differences were identified. This result may be attributed to three main factors. First, anesthesia management was tailored to each patient, with variations in the induction agents used, the duration of anesthesia, and the postoperative pain control methods used, which were not standardized. These factors may have contributed to the variability in recovery times. Second, the surgical techniques employed were not standardized, either. Both laparoscopic and open approaches were employed, potentially influencing complication rates and discharge times. Third, all of the patients who underwent surgery at our clinic were routinely transferred to the general surgery intensive care unit, and this transferal may have affected their CDC grades.

Globally, the prevalence of sarcopenia among individuals over 60 years of age is estimated to be approximately 10%. Sarcopenia is assessed based on criteria such as reduced muscle strength, decreased muscle mass or quality, and diminished physical performance [13]. It predominantly affects elderly individuals, and no significant difference between males and females has been reported [6]. However, contrary to the literature, our study found a statistically significant difference in the prevalence of sarcopenia between male and female patients (*p* < 0.05). This result may be due to our inclusion of patients undergoing colorectal cancer surgery, an association that is also consistent with the finding reported by Abotchie et al. [34], indicating that men over the age of 60 had a higher prevalence with respect to undergoing colorectal cancer surgery than women.

STAR was developed by Kara et al. as a tool for diagnosing sarcopenia [15]. In our study, the STAR value was compared with rectus femoris and vastus intermedius measurements, and VIT was found to be statistically significant. Salim et al. [3] demonstrated a correlation between USG-measured TMT and frailty or sarcopenia among elderly patients diagnosed with sarcopenia via CT. In our study, we examined total TMT, RFT, and VIT, and identified a statistically significant relationship between VIT and STAR values.

Sarcopenia is closely associated with poor postoperative outcomes and complications [35,36,37]. A major focus of our study was to evaluate the role of sarcopenia in postoperative recovery, complications, and discharge time. However, no statistically significant differences were found. This finding may be attributed to two main factors. First, the anesthesia protocol was individualized for each patient, resulting in variations in the induction agents used, the duration of anesthesia, and the postoperative pain management techniques employed, which were not uniform across cases. These discrepancies could have affected recovery times. Second, a combination of laparoscopic and open surgical techniques was used, rather than a single standardized approach; this fact may have influenced complication rates and discharge times.

In our study, RFT, VIT, and TMT were recorded using USG measurements. Subsequently, RFT/TL, VIT/TL, and TMT/TL values were calculated. No statistically significant differences were found regarding the impacts of these variables on postoperative recovery, complications, or discharge time, a result we attribute to the previously mentioned factors. The relationship between TMT/TL and frailty was found to be statistically significant, a finding consistent with previous studies [3,29,30,31].

Upon assessing the patients’ sarcopenia statuses using STAR values, and stratifying them by gender, we determined that, for sarcopenic males, a VIT/TL value below the cut-off of <0.041 (area under the curve (AUC): 0.808; 95% confidence interval (CI): 0.619–0.929; *p* = 0.0319) could assist in the diagnosis of sarcopenia with 92.3% sensitivity and 66.6% specificity. The inability to determine cut-off values for RFT/TL and TMT/TL measurements for sarcopenic males may be attributed to the insufficient homogeneity in the male patient distribution, the single-center nature of this study, and the fact that the study population consisted of patients undergoing colorectal cancer surgery.

For sarcopenic female patients, an RFT/TL value below the cut-off of <0.025 (AUC: 0.788; 95% CI: 0.481–0.958; *p* = 0.0391) was found to be useful in diagnosing sarcopenia with 62.5% sensitivity and 100% specificity. A VIT/TL value below <0.028 (AUC: 0.863; 95% CI: 0.565–0.986; *p* = 0.0009) was similarly found to assist in diagnosing sarcopenia with 62.5% sensitivity and 100% specificity. Furthermore, a TMT/TL value below <0.053 (AUC: 0.875; 95% CI: 0.579–0.989; *p*: 0.0002) was identified as useful for diagnosing sarcopenia with 62.5% sensitivity and 100% specificity. These measurements are considered cost-effective, rapid, easy to perform, and feasible for bedside application.

## 5. Limitations

The first limitation of our study is that it was conducted at a single center. The second limitation is that frailty and sarcopenia were evaluated exclusively among patients undergoing colorectal cancer surgery, excluding other surgical cases. The third limitation was the non-standardized anesthesia management, which varied among patients. Lastly, patient follow-ups were conducted only until they were discharged from the clinic, preventing the collection of sufficient data on long-term complications or mortality.

## 6. Conclusions

A key contribution of our study is the determination of cut-off values for diagnosing sarcopenia through USG measurements of TMT relative to TL. These values are believed to correlate with CT and MRI, which are considered the gold standards for sarcopenia diagnosis. Furthermore, USG measurements are cost-effective, bedside-applicable, and portable, and they do not exert the undesirable effects of radiation, making them practical and efficient diagnostic tools. However, there is a need for further studies with larger and more diverse patient populations to validate these findings.

In conclusion, our study found that male patients undergoing colorectal malignancy surgery were frailer and more sarcopenic. Frail and sarcopenic patients exhibited higher ASA scores. In addition, TMT measurements relative to TL, particularly VIT, were identified as valuable diagnostic parameters for sarcopenia.

## Figures and Tables

**Table 1:** FRAIL scoring system.

Scale Items		Score	
Fatigue	How often have you felt tired in the past four weeks?	Most of the time or all of the time = 1	Normal = 0
Resistance	By yourself and not using aids, can you climb one flight of stairs?	No = 1	Yes = 0
Ambulation	By yourself and not using aids, do you have any difficulty walking 200 m?	No = 1	Yes = 0
Illness	Do you have a history of hypertension, diabetes mellitus, chronic obstructive pulmonary disease, myocardial infarction, congestive heart failure, angina, asthma, arthritis, stroke, kidney disease, or cancer? (The score is based on whether the patient has 5 or more of these 11 conditions.)	Yes = 1	No = 0
Loss of weight	Have you lost more than 5% of your body weight in the past year?	Yes = 1	No = 0
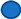 0 NORMAL 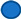 1–2 Pre-frail 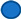 3–5 Frail Total …/5			

**Table 2:** Modified Aldrete Scoring System.

Scale Item		Score
Activity	Able to move all four extremities	2
	Able to move two extremities	1
	Unable to move extremities	0
Respiration	Able to breathe and cough	2
	Dyspneic or has restricted breathing	1
	Apneic	0
Circulation	Blood pressure within ±20% of pre-anesthetic level	2
	Blood pressure within ±20–49% of pre-anesthetic level	1
	Blood pressure within ±50% of pre-anesthetic level	0
Consciousness	Fully awake, oriented, and cooperative	2
	Responds to verbal stimuli	1
	No response	0
Oxygen saturation	SpO_2_ > 92% in room air	2
	Requires oxygen support to maintain SpO_2_ > 90%	1
	SpO_2_ < 90% despite oxygen support	0

SpO_2_: peripheral capillary oxygen saturation.

**Table 3:** Modified Clavien–Dindo Classification for surgical complications.

Grade	Complication
1	Normal postoperative follow-up that does not require pharmacological, surgical, or radiological intervention (use of antiemetics, antipyretics, analgesics, diuretics, physiotherapy, and electrolytes is acceptable; bedside wound infections are also considered within this category)
2	Complications requiring medical treatment beyond those listed in Grade 1 (blood transfusions, total parenteral nutrition (TPN), and antibiotic use are acceptable)
3A	Complications requiring intervention under regional/local anesthesia
3B	Complications requiring intervention under general anesthesia
4A	Complications leading to single-organ dysfunction requiring intensive care unit admission
4B	Complications leading to multiple organ failure
5	Patient death

**Table 4:** Demographic data pertaining to the patients.

Variable		Mean ± SD or *n* (%)
Age (years)		69.8 ± 7.26
Body mass index (kg/m^2^)		26.2 ± 4.77
Gender	Male	29 (69%)
	Female	13 (31%)
Frailty status	Not frail	6 (14%)
	Pre-frail	26 (62%)
	Frail	10 (24%)
Education level	No formal education	3 (7%)
	Primary school graduate	21 (50%)
	Middle school graduate	6 (14%)
	High school graduate	9 (21%)
	University graduate	3 (7%)
Marital status	Married	32 (76%)
	Widowed	10 (24%)
Sarcopenic status	Sarcopenic male	26 (62%)
	Non-sarcopenic male	3 (7%)
	Sarcopenic female	8 (19%)
	Non-sarcopenic female	5 (12%)

SD: standard deviation.

**Table 5:** Relationship between ASA scores and other variables.

		ASA I (n = 1)	ASA II (n = 18)	ASA III (n = 23)	*p*-Value
Age (years)		68.0 ± 0	66.9 ± 5.88	72.1 ± 7.68	0.0956
Weight (kg)		85.0 ± 0	71.2 ± 12.3	74.7 ± 15.7	0.4
Height (cm)		174 ± 0	166 ± 8.70	168 ± 10.4	0.438
Body mass index (kg/m^2^)		28.1 ± 0	25.8 ± 4.23	26.5 ± 5.31	0.786
Anesthesia recovery time		32.0 ± 0	34.9 ± 5.44	38.2 ± 7.15	0.265
CDC grade	1	0 (0%)	1 (6%)	1 (4%)	0.956
	2	1 (100%)	15 (83%)	16 (70%)	
	3A	0 (0%)	0 (0%)	0 (0%)	
	3B	0 (0%)	1 (6%)	1 (4%)	
	4A	0 (0%)	1 (6%)	4 (17%)	
	4B	0 (0%)	0 (0%)	0 (0%)	
	5	0 (0%)	0 (0%)	1 (4%)	
Discharge time (days)		6.00 ± 0	12.2 ± 17.2	10.5 ± 6.12	0.303
Sarcopenia	Present	1	14	19	0.954
	Absent	0	4	4	
Frailty status	Not frail	1	5	0	*p* < 0.05 *
	Pre-frail	0	12	14	
	Frail	0	1 *	9 *	

ASA: American Society of Anesthesiologists, and CDC: Clavien–Dindo Classification. * Statistically significant at *p* < 0.05.

**Table 6:** Relationships between frailty status and other variables.

		Not Frail	Pre-Frail	Frail	*p*-Value
Age		67.5 ± 4.09	68.1 ± 6.67	75.6 ± 7.62 *	*p* < 0.05 *
Gender	Male	4 (67%)	18 (69%)	7 (70%)	0.99
	Female	2 (33%)	8 (31%)	3 (30%)	
Body mass index		24.4 ± 1.97	27.1 ± 4.33	24.9 ± 6.55	0.135
Education level	No formal education	0 (0%)	2 (8%)	1 (10%)	0.893
	Primary school	2 (33%)	13 (50%)	6 (60%)	
	Middle school	1 (17%)	4 (15%)	1 (10%)	
	High school	2 (33%)	6 (23%)	1 (10%)	
	University	1 (17%)	1 (4%)	1 (10%)	
Marital status	Married	5 (83%)	22 (85%)	5 (50%)	0.083
	Widowed	1 (17%)	4 (15%)	5 (50%)	
Anesthesia recovery time		34.0 ± 4.94	35.5 ± 5.43	41.6 ± 8.38	0.056
Discharge time		7.17 ± 2.99	12.2 ± 14.5	10.8 ± 7.74	0.199
CDC grade	1	0 (0%)	2 (8%)	0 (0%)	0.244
	2	6 (100%)	20 (77%)	6 (60%)	
	3A	0 (0%)	0 (0%)	0 (0%)	
	3B	0 (0%)	2 (8%)	0 (0%)	
	4A	0 (0%)	2 (8%)	3 (30%)	
	4B	0 (0%)	0 (0%)	0 (0%)	
	5	0 (0%)	0 (0%)	1 (10%)	
Sarcopenia	Present	1 (3%)	14 (41%)	19 (56%)	
	Absent	0 (0%)	4 (50%)	4 (50%)	
Total muscle thickness		2.74 ± 0.413	2.79 ± 0.614 *	2.27 ± 0.446 *	*p* < 0.05 *

CDC: Clavien–Dindo Classification. * Statistically significant at *p* < 0.05.

**Table 7:** Relationships between sarcopenia status and other variables.

		Sarcopenic Female	Non-Sarcopenic Female	Sarcopenic Male	Non-Sarcopenic Male	*p*-Value
Age		65.0 ± 7.27 *	71.0 ± 8.51	71.9 ± 6.33 *	62.7 ± 4.62	*p* < 0.05 *
Body mass index		28.9 ± 4.25	24.2 ± 5.31	25.9 ± 4.52	25.2 ± 7.05	0.239
Education level	No formal education	2 (25%)	0 (0%)	1 (4%)	0 (0%)	0.839
	Primary school	4 (50%)	4 (80%)	11 (42%)	2 (67%)	
	Middle school	1 (12%)	0 (0%)	5 (19%)	0 (0%)	
	High school	0 (0%)	0 (0%)	8 (31%)	1 (33%)	
	University	1 (12%)	1 (20%)	1 (4%)	0 (0%)	
Marital status	Married	5 (62%)	3 (60%)	21 (81%)	3 (100%)	0.083
	Widowed	3 (38%)	2 (40%)	5 (19%)	0 (0%)	
Anesthesia recovery time		37.3 ± 4.30	37.8 ± 7.18	36.3 ± 7.60	35.3 ± 3.06	0.918
Discharge time		7.00 ± 1.93	22.0 ± 32.4	10.4 ± 6.10	10.0 ± 2.65	0.278
CDC grade	1	0 (0%)	1 (20%)	1 (4%)	0 (0%)	0.244
	2	8 (100%)	3 (60%)	18 (69%)	3 (100%)	
	3A	0 (0%)	0 (0%)	0 (0%)	0 (0%)	
	3B	0 (0%)	1 (20%)	1 (4%)	0 (0%)	
	4A	0 (0%)	0 (0%)	5 (19%)	0 (0%)	
	4B	0 (0%)	0 (0%)	0 (0%)	0 (0%)	
	5	0 (0%)	0 (0%)	1 (4%)	0 (0%)	

CDC: Clavien–Dindo Classification. * Statistically significant at *p* < 0.05.

## Data Availability

The data presented in this study are available on request from the corresponding author due to privacy reasons.

## Abbreviations

ASA	American Society of Anesthesiologists
FRAIL	Fatigue, Resistance, Ambulation, Illness, and Loss of Weight
RFT/TL	Rectus femoris thickness/thigh length
SD	Standard deviation
STAR	Sonographic Thigh Adjustment Ratio
TMT/TL	Total muscle thickness/thigh length
USG	Ultrasonography
VIT/TL	Vastus intermedius thickness/thigh length
BMI	Body mass index
CDC	Clavien–Dindo Classification
